# Prediction of Postprandial Blood Glucose Variability Using Machine Learning in Frequent Insulin Injection Therapy with a Simplified Carbohydrate Counting Model

**DOI:** 10.3390/nu17243832

**Published:** 2025-12-07

**Authors:** Hiroyuki Tominaga, Masahide Hamaguchi, Youji Hamaguchi, Ren Yashiki, Aki Yamaguchi, Tadaharu Arai, Masahiro Yamazaki, Noriyuki Kitagawa, Yoshitaka Hashimoto, Hiroshi Okada, Michiaki Fukui

**Affiliations:** 1 Department of Endocrinology and Metabolism, Graduate School of Medicine, Kyoto Prefectural University of Medicine, Kyoto 602-8566, Japan; htommy@koto.kpu-m.ac.jp (H.T.); mb221098@stu.kpu-m.ac.jp (R.Y.); nori-kgw@koto.kpu-m.ac.jp (N.K.); conti@koto.kpu-m.ac.jp (H.O.); michiaki@koto.kpu-m.ac.jp (M.F.); 2 S.I.P., Tokyo 141-0021, Japan; hamaguchi@sipartners.jp; 3 Persol Avc Technology Co., Ltd., Osaka 569-1194, Japan; yamaguchi.aki@persol-avct.co.jp (A.Y.); arai.tadaharu@persol-avct.co.jp (T.A.); 4 Department of Metabolism and Endocrinology, Japanese Red Cross Society Kyoto Daini Hospital, Kyoto 602-8026, Japan; masahiro@koto.kpu-m.ac.jp; 5 Department of Diabetes, Kameoka Municipal Hospital, Kameoka 621-8585, Japan; 6 Department of Diabetes and Endocrinology, Matsushita Memorial Hospital, Osaka 570-8540, Japan; y-hashi@koto.kpu-m.ac.jp

**Keywords:** diabetes, frequent insulin injection therapy, machine learning, blood glucose variability, carbohydrate counting method, transformer model

## Abstract

**Background/Objectives:** Postprandial glucose variability is a key challenge in diabetes management for patients receiving multiple daily insulin injections (MDI). This study evaluated transformer-based machine-learning models for predicting post-prandial glucose peaks and nadirs using pre-meal glucose, insulin dose, and nutritional input. **Methods:** In this observational study, 58 adults with diabetes provided dietary records, insulin logs, and continuous glucose monitoring data. After preprocessing and participant-level splitting (64:16:20), model-ready datasets comprised 6155/1449/1805 (train/validation/test) meal events for the Full-Nutrition model and 6299/1484/1849 for the Carbohydrate and Available-Carbohydrate models. We evaluated three transformer-based models and assessed performance using MAE, R^2^, and the Clarke error grid. **Results:** The Full Nutrition Model achieved MAEs of 32.2 mg/dL (peak) and 21.8 mg/dL (nadir) with R^2^ values of 0.58 for both. Carbohydrate-based models showed similar accuracy. Most predictions fell within Clarke error grid Zones A and B. **Conclusions:** Transformer-based machine-learning models can accurately predict postprandial glucose variability in MDI-treated patients. Carbohydrate-only inputs performed comparably to full-nutrient data, supporting the feasibility of simplified dietary inputs in clinical applications.

## 1. Introduction

The American Diabetes Association (ADA), the European Association for the Study of Diabetes (EASD), and the Japan Diabetes Society (JDS) emphasize the importance of reducing the development and progression of complications and maximizing patients’ quality of life (QOL) in the treatment of diabetic patients [1,2,3]. In particular, the ADA and EASD emphasize the importance of patient self-determination and quality of life in the management of blood glucose in patients with type 2 diabetes [4]. Minimizing glycemic variability—including postprandial excursions—is a contemporary clinical target supported by international consensus on CGM-derived metrics such as Time in Range and by recent outcome-focused reviews and meta-analyses [5,6,7,8]. In this context, many AI studies have focused on CGM forecasting and closed-loop pump algorithms. In contrast, our work targets a distinct, real-world need: **meal-specific** decision support for adults on **multiple daily injections (MDI)** who must determine prandial insulin doses themselves. To maintain blood glucose homeostasis within a certain range, insulin secretion is regulated to promote and inhibit insulin secretion. However, when insulin secretion is depleted or when endogenous insulin secretion alone is insufficient, external insulin replacement is required. Insulin replacement methods include subcutaneous injection with pen insulin or continuous subcutaneous injection using an insulin pump. These methods of administration must mimic the stimulation and suppression of insulin secretion in vivo, and pen insulin subcutaneous injection is used in combination with basal insulin and additional insulin in the form of frequent insulin injection therapy (MDI). In recent years, continuous insulin subcutaneous injection therapy has achieved blood glucose homeostasis by combining continuous glucose monitoring (CGM) and artificial intelligence (AI) to optimize insulin administration [9,10]. On the other hand, in many countries and regions, MDI is implemented with high frequency.

While many prior studies have applied AI to continuous glucose monitoring (CGM) forecasting, closed-loop insulin pump algorithms, or Food Insulin Index (FII)-based insulin adjustments, few have focused specifically on patients treated with multiple daily insulin injections (MDI). For example, Ahmed et al. developed a cloud-based deep learning model to forecast blood glucose trends from CGM data [11], while closed-loop automated insulin delivery (AID) systems continuously adjust insulin infusion via algorithms. Similarly, the FII approach, pioneered by Brand-Miller’s group, improved postprandial glucose control compared with carbohydrate counting in type 1 diabetes [12]. However, these approaches have predominantly been applied in pump users rather than MDI-treated patients. Notably, despite technological advances, MDI remains the most widely used insulin therapy worldwide due to its affordability and accessibility [13]. Indeed, pump penetration varies regionally, with adoption rates as low as 11–15% in some countries [14]. Consequently, the majority of insulin-treated patients—particularly adults and those in resource-limited settings—continue to rely on MDI. Recent high-impact AI studies have further highlighted this gap. Reinforcement-learning algorithms for insulin titration in hospitalized type 2 diabetes patients [15] and fully closed-loop systems eliminating mealtime boluses in type 2 adults [16] demonstrate the potential of AI in insulin management. Yet, these advances do not directly address the needs of MDI patients who must make meal-specific insulin dosing decisions on their own. Unlike CGM forecasting, which predicts near-future glucose trajectories, or closed-loop algorithms that automate insulin delivery, our approach aims to support meal-specific decision-making in MDI users. By predicting postprandial peak and nadir glucose responses from meal nutrient composition, pre-meal glucose, and insulin dose, our system addresses the practical and unique clinical question faced by MDI users: “Will this insulin dose adequately cover my blood sugar for this meal?” This distinction emphasizes the clinical context and real-world relevance of our study.

Recent advances in machine learning have enabled increasingly accurate prediction of postprandial blood glucose. Recurrent neural network (RNN)-based models, particularly long short-term memory (LSTM) architectures, remain central. Rabby et al. reported a root mean square error (RMSE) of 6.45 mg/dL at 30 min using a stacked LSTM with Kalman smoothing [17]. Other LSTM/GRU models have achieved RMSEs between 15 and 23 mg/dL. Shen and Kleinberg developed an incrementally retrained LSTM that transferred weights to new patients, reducing RMSE to approximately 10.2 mg/dL and achieving ≥97–99% of predictions in Clarke zones A/B [18]. Convolutional neural networks (CNNs) and hybrid models have further improved feature extraction. Li et al. developed a convolutional recurrent neural network (CNN–LSTM hybrid) and reported an RMSE of approximately 9.4 mg/dL over a 30 min horizon in simulated patients [19], while Ahmed et al. reported 5.93 mg/dL on a large dataset [20]. Mostafa et al. proposed a CNN–GRU ensemble for real-time IoT applications, which outperformed RNN-only models [21]. In type 2 diabetes, Ahmed et al. found that random forest models performed best, though with higher error (~31.9 mg/dL at 2 h), likely reflecting sparse data [20]. Transformer architectures are increasingly applied due to their ability to capture long-range dependencies. Bian et al. reported RMSEs of ~1.1–1.3 mg/dL using a Transformer–LSTM hybrid [22]. Moon et al. developed a BiT-MAML model combining bidirectional LSTM and Transformer layers, achieving ~24.9 mg/dL RMSE and >92% of predictions in Clarke zones A/B [23]. Patch-based variants, such as Crossformer and PatchTST, have reported RMSEs between 15 and 25 mg/dL depending on historical input length [24]. Meta-learning and transfer learning have emerged as promising strategies to address personalization and limited data. Moon’s BiT-MAML enabled rapid adaptation with small datasets [23]. Shen and Kleinberg’s IS-LSTM transferred weights across patients, lowering training demand and reducing error [18]. Deng et al. demonstrated that transfer learning with data augmentation improved performance, achieving >95% accuracy and 90% sensitivity within 1 h prediction horizons [25]. In summary, current deep learning models achieve 30 min RMSEs of ~6–25 mg/dL depending on data quality and personalization. Adaptive strategies such as transfer learning and meta-learning can further reduce error to ~10 mg/dL, with >90% of predictions falling within clinically safe Clarke zones. Hybrid and attention-based models, combined with adaptive training, currently offer the greatest promise for glucose prediction in MDI therapy. Beyond algorithm development, recent studies have expanded the application of AI in clinical diabetes care. Veluvali et al. demonstrated in a large-scale user study that an AI-enhanced CGM mobile application significantly improved time-in-range (TIR) and supported weight management, providing real-world evidence for the effectiveness of AI-based interventions [26]. Similarly, Brügger et al. applied machine learning models integrating CGM and dietary logs to predict individualized postprandial glucose excursions in Chinese patients with type 2 diabetes, highlighting both predictive accuracy and geographic validity [27]. Furthermore, Sheng provided a comprehensive review of AI applications in diabetes care, outlining current advances and future directions in screening, monitoring, and personalized therapy [28]. Taken together, these contemporary contributions, along with earlier foundational work, illustrate the rapidly evolving field of AI-based glucose prediction and position our study within this state-of-the-art landscape.

While MDI requires patients to self-determine mealtime insulin doses, this remains technically difficult. To support patients, we developed transformer-based machine-learning models that predict individual postprandial glucose peaks and nadirs based on pre-meal glucose, meal nutrients, and insulin dose. We evaluated their performance and compared three variants: the Full-Nutrition model, the Carbohydrate model, and the Available-Carbohydrate model.

## 2. Materials and Methods

### 2.1. Study Design and Participants

This was a multicenter observational study. Study subjects were participants in the multicenter, multipurpose, prospective cohort study/KAMOGAWA-DM cohort study of diabetic patients attending Kyoto Prefectural University of Medicine and related facilities. The study was approved by the Kyoto Prefectural University of Medicine Medical Ethics Review Committee (approval number: RBMR-E-466, 4 April 2013).

### 2.2. Data Collection

Participants recorded their meals using a standardized mobile dietary tracking application. Nutrient intake was extracted using a validated nutritional database. Blood glucose levels were continuously monitored using flash glucose monitoring systems (FreeStyle Libre; Abbott, Abbott Park, IL, USA). Insulin doses were self-reported by patients using standardized log sheets. Pre-meal glucose, postprandial peak glucose, and nadir values were extracted for each meal event. Step count and heart rate data were also collected via Apple Watch in a subset of participants. However, due to high rates of missing data, these variables were excluded from the final model inputs.

Pre-meal glucose source and selection. All participants wore FreeStyle Libre (Abbott); no SMBG values were used. The pre-meal glucose was defined as the nearest preceding Libre reading within 15 min of the logged meal time. If multiple readings existed, the closest preceding value was used; if no reading was taken within 30 min, the meal was excluded during preprocessing. To avoid temporal leakage and keep inputs practical for MDI users, no CGM history window (trajectory) was included—only this single scalar pre-meal value.

### 2.3. Data Preprocessing

All data were carefully preprocessed prior to model development to ensure accuracy and reproducibility.

First, missing data were handled as follows: step count and heart rate data collected via wearable devices exhibited a high proportion of missingness and were excluded from the final analysis. Participants with incomplete dietary records (n = 24) were excluded, leaving 58 participants. After preprocessing, the datasets contained the following numbers of meal events: Full-Nutrition (FN) 6155/1449/1805 (train/validation/test; total 9409), and Carbohydrate (CM)/Available-Carbohydrate (ACM) 6299/1484/1849 (train/validation/test; total 9632). The held-out test partitions therefore contained 1805 events for FN and 1849 for CM/ACM; the union of test meal events across models comprised 1888 unique events (defined by ID × pre-meal timestamp × mealtime). Physical activity variables. Step count and heart rate were collected via wearable devices in a subset; due to substantial missingness, these variables were excluded a priori from model inputs to prevent bias.

Second, to reduce bias and data leakage, dataset partitioning was performed at the participant level, with training, validation, and test sets split in a 64:16:20 ratio. This ensured that data from a given participant were not simultaneously present in multiple sets. To avoid contamination of the primary evaluation, all primary metrics were computed on an untouched holdout test set. For a separate exploratory personalization track, we created a duplicate of the test partition and, within each subject, split records into 20% adaptation/80% evaluation. This exploratory split does not affect the primary test results.

Third, continuous input variables (preprandial glucose, insulin dose, and nutrient features) were scaled using min–max normalization to the [0, 1] range prior to model training.

Finally, potential confounders were explored through subgroup analyses stratified by meal type, insulin dose category, and preprandial glucose level. Other possible confounders, such as physical activity and circadian variation, could not be assessed due to incomplete data and are acknowledged as study limitations.

### 2.4. Model Architecture

Rationale for simplified inputs. Our prespecified objective was to test whether carbohydrate-focused inputs—readily obtainable from food labels, barcode scanning, or menu databases—can achieve accuracy comparable to full-nutrient inputs while reducing patient logging burden in MDI therapy. “Available carbohydrate” was defined as total carbohydrate minus dietary fiber, reflecting the digestible fraction that directly contributes to postprandial glycemia. We constructed three transformer-based machine-learning models:

- **Full Nutrition Model:** All available macronutrient and micronutrient features were included.
- **Carbohydrate Model (CM):** The input was the total carbohydrate content of each meal, as reported by the nutritional database, including starch, sugars, and dietary fiber.
- **Available Carbohydrate Model (ACM):** The input was restricted to digestible carbohydrates—starch and sugars—excluding dietary fiber, which is not absorbed and therefore does not directly contribute to postprandial glycemia.

This distinction reflects the concept of “available carbohydrate” used in calculating glycemic load.

Each model consisted of two transformer encoder layers with multi-head self-attention, an embedding dimension of 64, and positional encoding. The input features included preprandial glucose level, prandial insulin dose, and the relevant nutritional variables.

Each transformer encoder block included:

- A multi-head self-attention mechanism with 8 attention heads;
- A position-wise feed-forward network employing the ReLU activation function;
- **Bias terms** in all linear layers;
- **Dropout layers** (rate = 0.3) applied after both the attention and feed-forward sublayers;
- **Layer normalization** after each sublayer.

These details enhance reproducibility and clarify how the models were regularized during training.

Thus, while CM reflects the total carbohydrate entry typically shown on food labels, ACM corresponds to the physiologically active portion that raises blood glucose.

Unlike Ref. [29], which evaluates 30-min CGM forecasting with imputation/smoothing on an insulin-pump dataset, our task is meal-specific peak and nadir prediction in MDI users using meal-level features only (no CGM history windows).

### 2.5. Model Training

Data were randomly split at the participant level into training (64%), validation (16%), and test (20%) sets to prevent data leakage. For the main evaluation, the test set was held out entirely and never used for model adjustment.

Training employed mean squared error loss and the Adam optimizer (learning rate = 0.001) with a batch size of 32. Early stopping (patience = 10 epochs) was applied based on validation loss.

**Figure A1:**
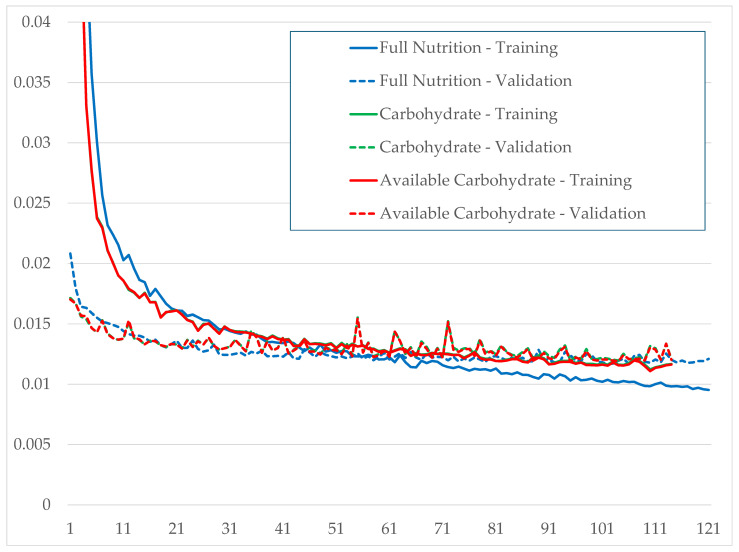
Training and validation loss curves for the three transformer models (Full Nutrition, Carbohydrate, Available Carbohydrate) on the breakfast subset across all participants.

To illustrate the learning process, we plotted the training and validation loss curves for each of the three models (Full Nutrition, Carbohydrate, and Available Carbohydrate); these are presented in Figure A1 and demonstrate stable convergence with early stopping preventing overfitting.

### 2.6. Evaluation Metrics and Definitions

Evaluation Metrics:

Primary metrics: Mean absolute error (MAE), coefficient of determination (R^2^), Pearson correlation, and Clarke error grid (clinical acceptability).

Exploratory metrics: Postprandial glucose excursion (PPGE) and approximate area under the curve (AUC). These were exploratory only, did not change conclusions, and are available on request; they are not summarized in the Abstract.

Definitions:

MAE: Mean absolute difference between predicted and actual values.

R^2^: Proportion of variance explained.

Pearson r: Linear correlation between predicted and actual values.

Clarke error grid: Clinical accuracy zones (A–E).

PPGE: Postprandial peak glucose minus pre-meal glucose.

Approx. AUC: Mean of upward and downward deviations from pre-meal glucose.

### 2.7. Statistical Analysis

All analyses were performed using Python 3.10. Numerical data were processed using Pandas (v1.5) and NumPy (v1.23.1). Model training and evaluation used PyTorch (v1.9) and Scikitlearn(v1.1). Graphical visualizations were generated with Matplotlib (v3.5.2) and Seaborn (v0.11.2). Normality of continuous variables was tested using the Shapiro–Wilk test. Differences between subgroups were evaluated using one-way ANOVA. A *p*-value of < 0.05 was considered statistically significant.

## 3. Results

### 3.1. Participant Characteristics

**Figure 1:**
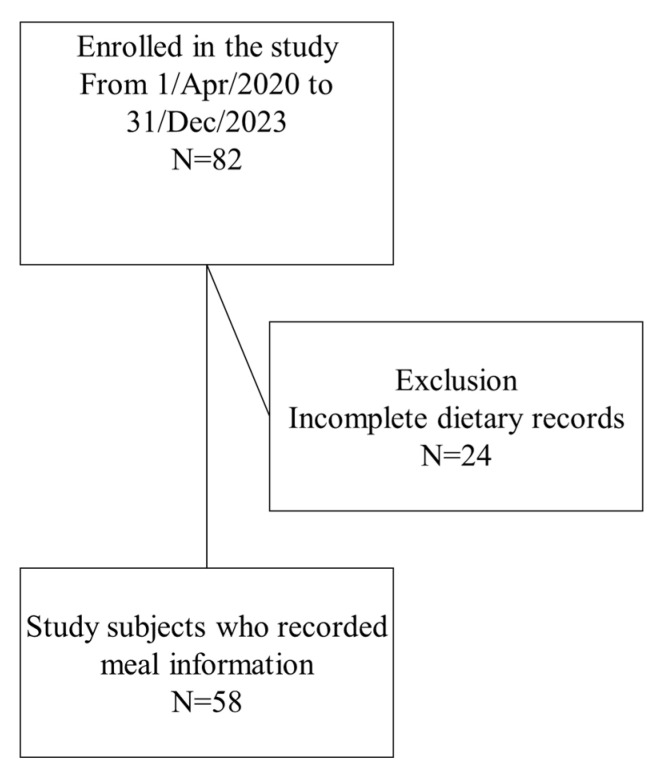
Flow chart of study subject enrollment and analysis.

A total of 82 individuals with diabetes were enrolled in this study between 1 April 2020, and 31 December 2023 (Figure 1). This observational study was conducted across multiple centers (Kyoto Prefectural University of Medicine and collaborating hospitals). Of the enrolled participants, 24 were excluded due to incomplete dietary records. The remaining 58 participants provided complete data on dietary intake, pre- and postprandial blood glucose levels, and insulin dose logs, and were included in the final analysis. After preprocessing and participant-level splitting (64:16:20), the **held-out test set** comprised **1888 unique meal events** across models, of which **1805** were available for the Full-Nutrition model and **1849** for each of the Carbohydrate and Available-Carbohydrate models. Table 1 summarizes their baseline characteristics, including age, diabetes type, treatment modalities, insulin regimen, and complication status. Ethical approval was obtained from the institutional review board.

### 3.2. Overall Model Performance

**Figure 2:**
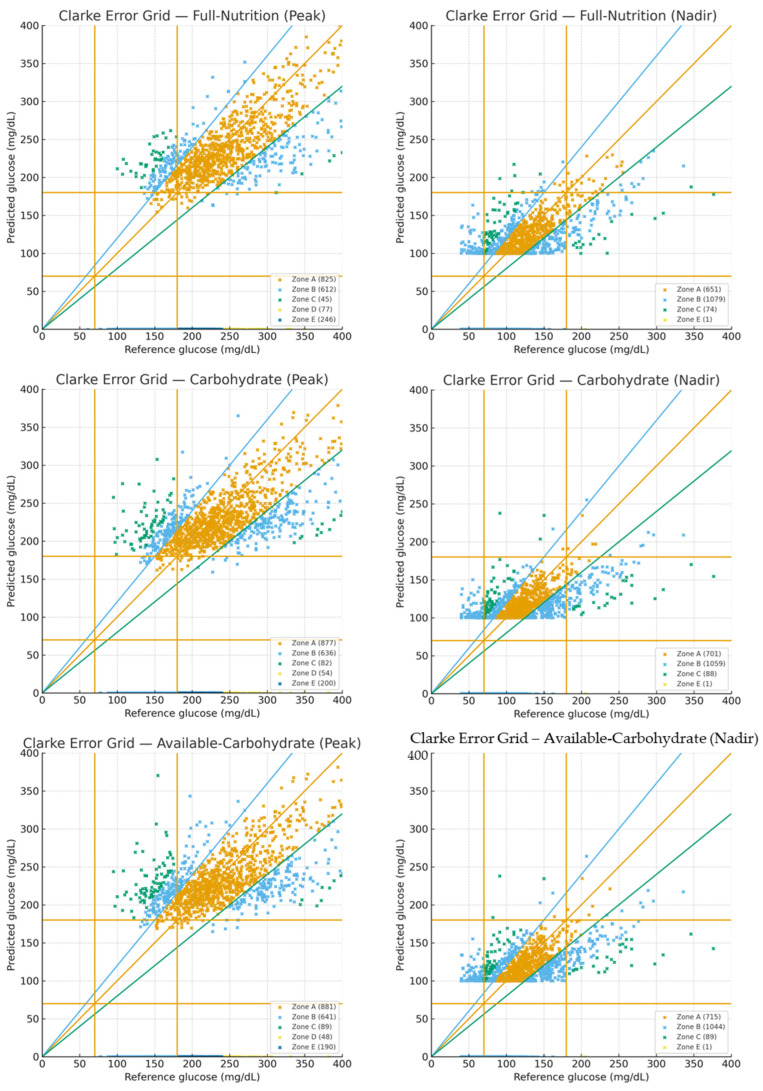
**Clarke error grid analysis of postprandial glucose predictions.** Panels show peak (**top row**) and nadir (**bottom row**) across three models: Full-Nutrition (FN), Carbohydrate (CM), and Available-Carbohydrate (ACM). Only held-out test predictions are shown. The number of plotted points per plot is 1805 for FN and 1849 for both CM and ACM; thus, per outcome (row) the total is 5503 points and 11,006 across both outcomes. Points are colored by Clarke error zones: Zone A (clinically accurate), Zone B (benign), Zone C (overcorrection), Zone D (failure to detect hypo/hyperglycemia), and Zone E (erroneous decisions). Reference lines include the identity line (y = x), ±20% lines, and glucose thresholds at 70 and 180 mg/dL. Across models, most points fall within Zones A/B, consistent with clinical acceptability.

Three machine-learning models were evaluated: Full Nutrition Model, Carbohydrate Model, and Available Carbohydrate Model. Clinical accuracy was further assessed using Clarke Error Grid Analysis. Figure 2 displays Clarke error grids for **both outcomes** (peak and nadir) across **all three models**. Table 2 summarizes Clarke outcomes on the held-out test set. For **peaks**, A + B proportions were **79.6% (FN)**, **81.9% (CM)**, and **82.3% (ACM)**; for **nadirs**, A + B proportions were **95.8% (FN)**, **95.2% (CM)**, and **95.1% (ACM)**, indicating high clinical acceptability across input strategies.

#### 3.2.1. Bland–Altman Analysis

**Figure 3:**
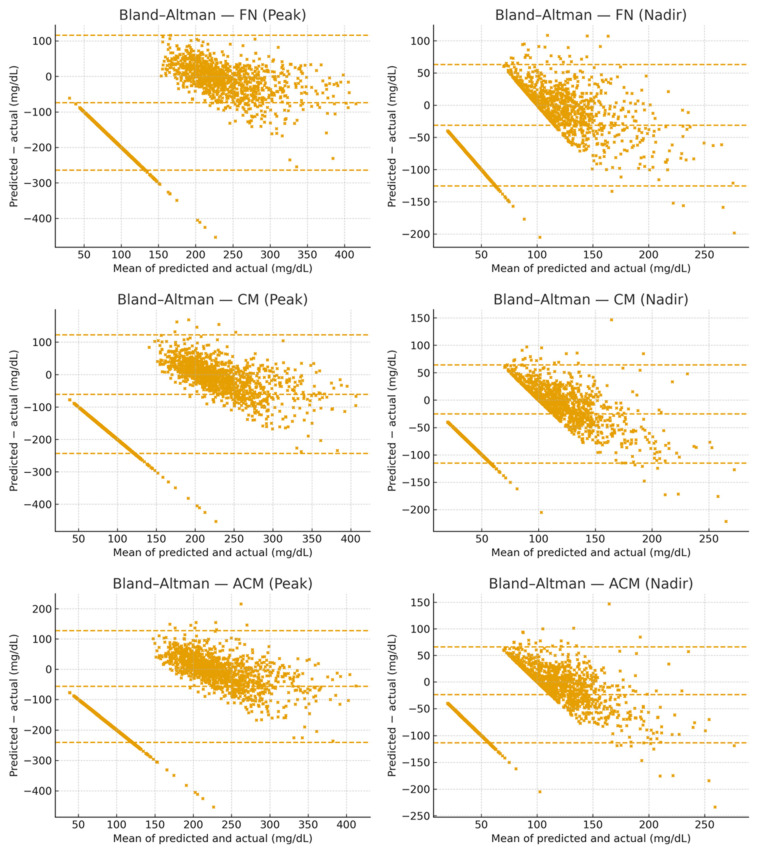
**Bland–Altman plots for postprandial glucose predictions.** Panels show peak (**top row**) and nadir (**bottom row**) across Full-Nutrition (FN), Carbohydrate (CM), and Available-Carbohydrate (ACM) models. The *y*-axis shows (*predicted − actual*); the middle-dashed line indicates the mean bias, and the upper and lower dashed lines indicate the 95% limits of agreement (LOA = bias ± 1.96 × SD of the differences). Peak: FN: bias = −1.83 mg/dL; LOA = [−102.40, 98.74] mg/dL, CM: bias = −2.27 mg/dL; LOA = [−106.35, 101.81] mg/dL, ACM: bias = −1.69 mg/dL; LOA = [−105.77, 102.38] mg/dL. Nadir: FN: bias = +0.47 mg/dL; LOA = [−48.72, 49.66] mg/dL, CM: bias = +0.53 mg/dL; LOA = [−50.24, 51.30] mg/dL, ACM: bias = +0.57 mg/dL; LOA = [−50.24, 51.38] mg/dL. Numerical reporting replaces subjective descriptors; interpretation aligns with the Clarke grid results in Figure 2.

Bland–Altman plots (Figure 3) indicated **near-zero mean bias** for both outcomes and **similar 95% limits of agreement (LOA)** across input strategies. For **peaks**, the mean bias ranged from **−2.27 to −1.69 mg/dL**, with LOA of approximately **−106 to +102 mg/dL** depending on the model. For **nadirs**, the mean bias ranged from **+0.47 to +0.57 mg/dL**, with LOA of approximately **−50 to +51 mg/dL**. These results, considered together with the Clarke grids (Figure 2), support **clinical acceptability** (predominantly Zones A/B) and show **comparable performance** across Full-Nutrition, Carbohydrate, and Available-Carbohydrate models.

#### 3.2.2. Prediction Error by Mean Absolute Error (MAE)

The mean absolute error (MAE) was used to evaluate the deviation between predicted and actual glucose values for each model. For peak glucose predictions, the Full Nutrition Model showed an MAE of 32.23 mg/dL, the Carbohydrate Model 33.02 mg/dL, and the Available Carbohydrate Model 32.98 mg/dL. For nadir glucose predictions, the MAEs were 21.76 mg/dL, 21.60 mg/dL, and 21.73 mg/dL, respectively. These results indicate that all three models provided comparable prediction accuracy for both peak and nadir values, with only minor differences in MAE across input strategies. The use of total carbohydrate or digestible carbohydrate alone did not substantially diminish predictive performance relative to the more complex full-nutrient input, suggesting that simpler input models may be sufficient for clinical application. Across input strategies, absolute MAE differences were ≤0.8 mg/dL for peak (32.23 vs. 33.02 vs. 32.98) and ≤0.2 mg/dL for nadir (21.76 vs. 21.60 vs. 21.73), indicating negligible practical differences within this cohort.

#### 3.2.3. Goodness-of-Fit Evaluation by R^2^ Score

The coefficient of determination (R^2^) was used to assess the proportion of variance in actual glucose values explained the transformer-based machine-learning models. For peak glucose prediction, the Full Nutrition Model achieved an R^2^ of 0.58, while the Carbohydrate and Available Carbohydrate Models both yielded R^2^ scores of 0.56. For nadir glucose prediction, R^2^ was also 0.58 in the Full Nutrition Model and 0.56 in both simplified models. These findings are consistent with the MAE results and suggest that all three models provided similarly strong performance in explaining the variation in postprandial glucose levels. Notably, the simplified input models based solely on carbohydrate and digestible carbohydrate performed nearly as well as the Full Nutrition Model, reinforcing their potential for practical clinical application.

### 3.3. Subgroup Analysis

#### 3.3.1. Subgroup Analysis by Meal Type, Insulin Dose, and Pre-Meal Glucose

**Figure 4:**
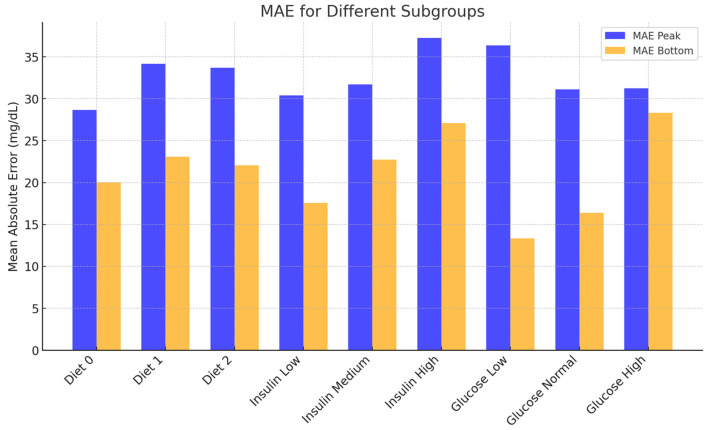
Mean absolute error (MAE) of postprandial glucose predictions stratified by meal type, insulin dose category, and pre-meal glucose level. Panels display the MAE for peak and nadir glucose predictions stratified by meal type (breakfast, lunch, dinner), insulin dose category (low < 2 U, medium 2–5 U, high > 5 U), and pre-meal glucose level (low < 100 mg/dL, medium 100–140 mg/dL, high > 140 mg/dL). MAEs were generally lowest in the breakfast subgroup and in those receiving medium-dose prandial insulin. Higher errors were observed for lunch and high-dose insulin groups, reflecting greater postprandial variability.

To evaluate the influence of clinical context on model performance, subgroup analyses were conducted using 3 × 3 × 3 combinations of meal type, insulin dose category, and pre-meal glucose category. As shown in Figure 4, prediction accuracy varied across subgroups. MAEs were generally lower for breakfast meals and moderate insulin doses, suggesting more predictable glycemic responses under these conditions.

Conversely, higher MAEs were observed in the lunch and high-dose insulin subgroups, possibly due to greater inter-individual variability or more complex metabolic dynamics during daytime hours. Pre-meal glucose level also showed a modest association with prediction error, with slightly improved performance in the medium glucose range (100–140 mg/dL).

These findings indicate that model performance is robust across a range of clinical scenarios, though prediction accuracy may vary with physiologic and behavioral factors.

#### 3.3.2. R^2^ Scores Across Clinical Subgroups

**Figure 5:**
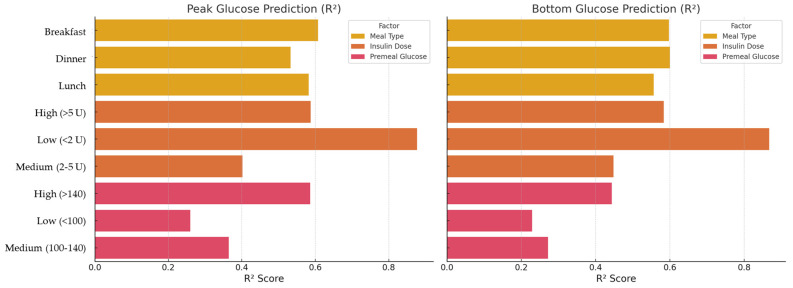
**Coefficient of determination (R^2^) for postprandial glucose predictions stratified by meal type, insulin dose category, and pre-meal glucose level.** Bar plots show R^2^ scores for peak (**left panel**) and nadir (**right panel**) glucose predictions across nine independent clinical subgroups categorized by meal type (breakfast, lunch, dinner), insulin dose (low < 2 U, medium 2–5 U, high > 5 U), and pre-meal glucose level (low < 100 mg/dL, medium 100–140 mg/dL, high > 140 mg/dL). Higher R^2^ scores indicate stronger agreement between model-predicted and actual glucose values.

Figure 5 presents R^2^ scores for peak and nadir glucose predictions across nine independent clinical subgroups defined by meal type, insulin dose, and pre-meal glucose level.

Among the meal types, breakfast consistently showed the highest R^2^, reflecting more stable postprandial glucose dynamics in the morning. Medium-dose insulin (2–5 U) achieved the best model fit, suggesting that moderate insulin administration yields more predictable responses compared to low or high doses.

For pre-meal glucose levels, the medium range (100–140 mg/dL) demonstrated the most accurate predictions, while extreme low or high glucose states showed lower R^2^ values.

Overall, the analysis indicates that model performance is most reliable under physiologically moderate conditions and highlights subgroups where predictive accuracy could be further optimized.

## 4. Discussion

### 4.1. Summary of Main Findings

In this study, we developed and validated transformer-based machine-learning models to predict postprandial blood glucose variability in individuals receiving multiple daily injection (MDI) therapy. Our three models—a Full Nutrition Model incorporating all macronutrient and micronutrient inputs, a Carbohydrate Model using total carbohydrates, and an Available Carbohydrate Model limited to digestible carbohydrates—demonstrated comparable accuracy for predicting postprandial glucose peaks and nadirs. Importantly, the simplified carbohydrate-based models performed nearly as well as the more complex full-nutrient model, suggesting that detailed nutrient entry may not be essential for clinically meaningful prediction. From a usability standpoint, carbohydrate-only (or available-carbohydrate) entry substantially reduces logging burden compared with full macronutrient/micronutrient input while maintaining comparable accuracy in this cohort. In practice, carbohydrate estimation can be further streamlined by barcode lookup, menu databases, or photo-based portion tools, enhancing feasibility for real-world MDI users. Our findings support a **low-burden pathway** to meal-level decision support in MDI therapy: **carbohydrate-only inputs** achieved accuracy **comparable** to full-nutrient inputs (peak MAE differences ≤ 0.8 mg/dL; nadir ≤ 0.2 mg/dL), suggesting that detailed macro/micronutrient logging is **not essential** for clinically meaningful prediction in this cohort. This aligns with real-world workflows (labels/barcodes/menu databases) and may facilitate adoption without the overhead of comprehensive nutrient entry.

### 4.2. Context and Comparison with Prior Work

Previous studies have developed AI- and model-based systems for glucose prediction, most often in the context of CGM forecasting or closed-loop pump algorithms. For example, Cobelli et al. reported that LSTM-based predictors achieved mean absolute errors (MAE) of 8–36 mg/dL across 15–90 min horizons in diverse populations [30]. Other multi-scale LSTM approaches reported RMSE values of 19.0 and 32.0 mg/dL at 30 and 60 min, corresponding to MAEs of ~13.5 and 23.8 mg/dL [29]. These results are consistent with reviews noting that short-term glucose predictions generally achieve MAEs in the range of 25 to 40 mg/dL, albeit with variability across patients [31]. Mechanistic and hybrid models have also contributed substantially. A recent universal minimal model demonstrated strong agreement with reference glucose–insulin dynamics (R^2^ ≈ 0.99), underscoring the predictive potential of physiological approaches [32]. Decision support systems (DSS) for insulin dosing extend these methods to clinical application. Tyler et al. developed a k-nearest-neighbor DSS for type 1 diabetes, achieving expert-level agreement and improving simulated time-in-range from ~60% to ~80% [33]. Within this context, our transformer-based models achieved MAEs of ~32 mg/dL (peak) and ~22 mg/dL (nadir), with R^2^ ≈ 0.56–0.58, demonstrating performance consistent with or slightly better than prior reports, while specifically targeting real-world MDI therapy patients. From a clinical perspective, the observed MAEs of ~32 mg/dL for postprandial peaks and ~22 mg/dL for nadirs warrant consideration regarding their acceptability for safe insulin decision support. Prior AI- and model-based glucose prediction studies typically report MAEs ranging from 25 to 40 mg/dL, depending on prediction horizon and patient population [29,33]. In this context, our model performance is comparable to or slightly better than established benchmarks. Moreover, Clarke error grid analysis in our study confirmed that the vast majority of predictions fell within Zones A and B, indicating clinical safety. While such error margins may not yet justify fully automated insulin dosing, they are sufficient for supportive applications designed to aid patients in estimating insulin doses and anticipating risky glycemic excursions. Thus, the present results highlight the feasibility of machine-learning-assisted glucose prediction as a step toward practical, patient-centered decision support in MDI therapy.

Previous studies have explored machine learning-driven glucose prediction, but most have focused on continuous glucose monitoring (CGM)-based forecasting or closed-loop insulin pump algorithms, rather than supporting patients on MDI therapy. Tyler et al. [33] proposed an AI-driven decision support tool for insulin dosing in type 1 diabetes, while Jödicke et al. [34] employed sparse nonlinear modeling to predict glucose dynamics. Similarly, Ng et al. [35] demonstrated a physiologic minimal model for glucose homeostasis. Our work builds on these efforts by leveraging transformer architectures—recently applied in other biomedical domains—and tailoring them for meal-specific postprandial prediction in MDI patients.

### 4.3. Model Performance and Clinical Acceptability

The models achieved mean absolute errors (MAEs) of ~32 mg/dL for peak glucose and ~22 mg/dL for nadirs across all three input strategies, with R^2^ values consistently around 0.56–0.58. These findings indicate that our machine-learning models can explain a substantial proportion of postprandial glycemic variability in real-world MDI therapy. Notably, using only carbohydrate or available carbohydrate data yielded prediction performance nearly identical to the full nutrient model. This suggests that carbohydrate content is the dominant determinant of postprandial glycemic excursions and that simplified input models—requiring less patient effort—may be sufficient for practical use. Clarke Error Grid analysis confirmed the clinical safety of predictions: most values fell within Zone A (clinically accurate) or Zone B (benign error), while very few points appeared in Zones D or E, which would represent dangerous prediction errors. Bland–Altman analysis further demonstrated minimal systemic bias, supporting the models’ robustness across the observed glucose range.

### 4.4. Subgroup Findings

Subgroup analyses revealed that prediction accuracy was highest for breakfast meals and moderate insulin doses (2–5 U), while lunch and high-dose insulin groups showed greater variability. Predictions were also most reliable in the medium pre-meal glucose range (100–140 mg/dL). These trends likely reflect physiologic stability under moderate conditions and more complex metabolic dynamics during lunch and with higher insulin requirements. In particular, breakfast may be more predictable because it follows overnight fasting and is less affected by residual nutrients or physical activity, and meal composition tends to be relatively standardized. Similarly, moderate insulin doses are often administered for meals of moderate size, where insulin action and glycemic load are better balanced, whereas higher doses are usually required for larger or mixed-nutrient meals, which introduce greater variability. Such insights can inform model refinement, potentially enabling adaptive prediction strategies that account for meal timing, dosing patterns, and baseline glucose state.

### 4.5. Clinical Implications and Feasibility

The findings have meaningful implications for patient self-management support. Carbohydrate counting remains the cornerstone of prandial insulin adjustment [36,37,38,39], yet many patients struggle with the complexity of estimating carbohydrate content [40,41,42]. Our study suggests that machine learning can deliver accurate postprandial glucose predictions—even with simplified inputs—potentially reducing the cognitive burden on patients while improving safety. Such models could be integrated into decision support tools or smartphone applications, offering real-time feedback to patients and clinicians.

### 4.6. Role of Nutrients Beyond Carbohydrate

Previous studies have shown that meal composition has profound effects on postprandial glucose responses. Bell et al. demonstrated that fat, protein, and glycemic index independently affect postprandial glucose and can cause delayed hyperglycemia [43]. Neu et al. emphasized that high-fat and high-protein meals require insulin adjustments beyond standard carbohydrate-based bolus dosing [44]. Wolever et al. established the concept of the glycemic index, showing that identical carbohydrate loads can produce different glucose responses depending on food quality [45]. Nimri et al. compared the Pankowska equation with the Food Insulin Index for adjusting insulin for fat and protein, finding that while these methods may reduce late postprandial hyperglycemia, they also increase the risk of hypoglycemia [46]. These findings underscore the conceptual rationale for integrating multi-nutrient data into prediction models, even if carbohydrate remains the dominant determinant of postprandial glycemia.

### 4.7. Diabetes Technology Context

Our work should also be viewed alongside rapid advances in diabetes technology. Giuseppe Scidà et al. reported that hybrid closed-loop (HCL) systems have significantly improved glycemic control in people with type 1 diabetes but still struggle to completely prevent postprandial spikes [47]. Bergenstal et al. demonstrated that next-generation automated systems can further reduce hyperglycemia without increasing hypoglycemia, yet these systems remain dependent on carbohydrate announcements and are not fully automated [48]. Scidà et al. provided international consensus and evidence that continuous glucose monitoring (CGM) is central to diabetes management and has transformed how glucose data are interpreted [47]. Our model’s ability to predict meal responses within MDI therapy complements these technologies, potentially bridging the gap between manual bolus decisions and automated systems.

Liu K et al. reviewed machine learning-based glucose prediction models, noting that short-term forecasts can achieve good accuracy but remain challenged by real-world variability and the integration of meal and insulin data [49]. Pinsker et al. emphasized that the next generation of artificial pancreas systems will depend on increasingly sophisticated glucose prediction algorithms to approach full automation [50]. By leveraging real-world dietary, insulin, and CGM data, our transformer-based model aligns with this vision, contributing to the broader effort to enhance prediction accuracy and support future decision support tools and fully automated insulin delivery systems.

### 4.8. Generalizability and Future Directions

Overall, our findings illustrate both the potential and the current limitations of machine-learning-driven glucose prediction. The primary aim of this study was to examine the feasibility of simplified nutritional inputs for clinical application, since carbohydrate counting remains the cornerstone of prandial insulin adjustment but is often burdensome for patients in daily life. Our results demonstrated that carbohydrate-only models achieved nearly identical predictive accuracy compared with the more complex full-nutrient model, thereby providing proof-of-concept that simplified input requirements could reduce cognitive burden while maintaining clinical utility for patients on MDI therapy.

At the same time, prior research has established that dietary fat and protein substantially affect postprandial glucose excursions, often inducing delayed hyperglycemia [43,44,45,46]. In our cohort, however, the inclusion of fat and protein in the Full Nutrition Model did not confer a measurable predictive advantage over carbohydrate-based models, likely reflecting the predominance of balanced Japanese-style meals with moderate macronutrient distribution. Thus, carbohydrate emerged as the dominant determinant of postprandial glycemia under these conditions. Given the modest sample size, we did not fit additional mixed nutrient models (e.g., carbohydrate + fat/protein) to avoid multiple testing and overfitting; testing such hybrids is a prespecified priority for future, larger multi-center cohorts.

Nevertheless, this finding should not be interpreted as diminishing the importance of fat and protein in other dietary contexts. Particularly in Western-style or high-fat/high-protein meals, delayed glycemia remains clinically relevant, and future research should explicitly test hybrid nutrient models (e.g., carbohydrate + fat/protein) in larger and more diverse cohorts. Such efforts will be essential to improve generalizability, refine predictive performance, and ensure that machine-learning-based decision support systems can adapt to diverse dietary environments. Building upon the present proof-of-concept, these future directions may further enhance the clinical utility of AI tools for supporting real-world insulin dosing decisions in MDI users.

### 4.9. Limitations

This study has several limitations. First, the cohort size was 58 participants from a single regional network, which may limit generalizability. Second, while carbohydrate-only inputs reduce logging burden relative to full nutrient entry, our approach still requires basic diet entry; integration with barcode/menu databases or photo-based tools could further reduce burden. Third, using a single pre-meal Libre value simplifies inputs and avoids temporal leakage but may be sensitive to sensor noise; sensitivity analyses (e.g., median within −15–0 min) are warranted. Finally, the models predict glucose responses but do not output insulin doses; translating predictions into dosing advice will require additional development, validation, and regulatory evaluation. Future work should extend validation to larger, multi-center MDI cohorts, test mixed-nutrient models (e.g., carbohydrate + fat/protein), and prospectively evaluate personalization strategies (e.g., subject-specific adaptation) using pre-specified protocols. In this initial study, we prioritized an untouched participant-level hold-out test set as the primary evaluation to provide an unbiased out-of-sample estimate and to prevent subject-level leakage. Grouped K-fold cross-validation at the participant level is a useful complementary within-cohort robustness check and will be incorporated alongside prospective external validation in future work.

## 5. Conclusions

In conclusion, this study demonstrates that transformer-based machine-learning models can predict postprandial glucose excursions in patients using multiple daily injections with clinically acceptable accuracy. Importantly, simplified carbohydrate-only input models performed nearly as well as full-nutrition models, highlighting their potential practicality for real-world use where detailed nutrient logging is burdensome. Such models could complement, rather than replace, closed-loop insulin delivery systems by providing meal-specific guidance for the large population of patients who remain on injection therapy. Nonetheless, our findings are based on a multicenter dataset from a single regional network with a modest sample size, and broader external validation is needed. Future research should pursue larger, multi-center studies to confirm generalizability, integrate more comprehensive lifestyle factors such as exercise and sleep, and ultimately translate predictive accuracy into safe and effective insulin dosing support.

## Figures and Tables

**Table 1:** (**a**) Clinical and Therapy Characteristics. (**b**) Demographic and Laboratory.

(**a**)			
**Variable**	**Number of Subjects**	**Medication**	**Number of Subjects**
Male	27	Biguanide	4
Female	31	SGLT2i	10
Type1	46	Glinide	1
Type2	9	Alpha GI	4
Steroid	3	GLP-1RA	9
Smoking status		ARB	13
Never	36	ACEi	2
Former	14	CCB	12
Current	8	Diuretics	2
Alcohol consumption	30	Alpha blocker	2
Exercise	6	βblocker	2
Nephropathy		MRA	2
micro albuminuria phase	6	Statin	19
proteinuria phase	6	Fibrate	2
renal failure phase	3		Mean ± SD
Retinopathy		Bolus insulin dose, morning	7.86 ± 3.61
simple retinopathy	3	Bolus insulin dose, lunch	8.08 ± 3.46
pre-proliferative retinopathy	5	Bolus insulin dose, evening	7.97 ± 3.57
proliferative retinopathy	5	Basal insulin dose	12.75 ± 6.79
Neuropathy	8	GLP-1RA dose	0.57 ± 2.06
(**b**)			
**Measurement**	**Mean ± SD**	**Measurement**	**Mean ± SD**
Age, years	57.0 ± 12.31	Aspartate Aminotransferase (AST) (U/L)	23.29 ± 13.33
Body weight at 20 years old, kg	67.85 ± 19.56	Alanine Aminotransferase (ALT) (U/L)	21.34 ± 18.26
Maximum body weight, kg	75.66 ± 24.28	γ-Glutamyl Transpeptidase (U/L)	24.26 ± 18.92
Age at maximum body weight, years	35.44 ± 16.9	Alkaline Phosphatase (U/L)	205.16 ± 111.3
Current body weight, kg	61.93 ± 12.4	Lactate dehydrogenase (U/L)	191.17 ± 37.38
Height, cm	162.28 ± 8.29	Creatine Kinase (U/L)	133.45 ± 49.17
systolic blood pressure, mmHg	131.75 ± 16.81	Total bilirubin(mg/dL)	0.73 ± 0.31
diastolic blood pressure, mmHg	75.04 ± 16.37	Albumin (g/dL)	4.14 ± 0.41
Heart rate, bpm	81.33 ± 11.94	Blood glucose(mg/dL)	174.83 ± 69.81
White Blood Cell Count (WBC) (×10^3^/μL)	6.12 ± 1.55	Serum C peptide	0.7 ± 1.27
Red Blood Cell Count (RBC) (×10^6^/μL)	45.75 ± 6.57	Anti GAD antibody	25.28 ± 100.97
Hemoglobin (g/dL)	13.96 ± 1.45	Hemoglobin A1c (%)	7.79 ± 0.88
Hematocrit (%)	41.99 ± 4.28	Total cholesterol(mg/dL)	206.6 ± 35.56
Platelet Count (×10^3^/μL)	180.27 ± 109.83	High-Density Lipoprotein Cholesterol (mg/dL)	71.84 ± 19.84
Urine pH	6.07 ± 0.78	Low-Density Lipoprotein Cholesterol (mg/dL)	115.04 ± 33.61
Urine ketone body	−0.72 ± 0.45	Triglycerides (mg/dL)	135.52 ± 79.32
Urine microalbumin (mg/dL)	967.25 ± 2431.88	Uric Acid (mg/dL)	4.8 ± 1.34
Urine Creatinine(mg/dL)	61.6 ± 31.75	Blood Urea Nitrogen (mg/dL)	16.71 ± 5.42
UACR (mg/gCr)	635.84 ± 1218.24	Sodium (mEq/L)	140.75 ± 1.69
Creatinine(mg/dL)	0.84 ± 0.4	Potassium (mEq/L)	4.41 ± 0.34
Estimated Glomerular Filtration Rate (mL/min/1.73 m^2^)	71.69 ± 20.43	Chloride (mEq/L)	103.56 ± 2.94

Values are presented as number of subjects or mean ± standard deviation (SD). Type 1, type 2: type of diabetes. ARB: angiotensin II receptor blocker; ACEi: angiotensin-converting enzyme inhibitor; CCB: calcium channel blocker; MRA: mineralocorticoid receptor antagonist; GLP-1RA: glucagon-like peptide-1 receptor agonist; SGLT2i: sodium-glucose cotransporter-2 inhibitor; Alpha GI: alpha-glucosidase inhibitor, UACR: Urinary Albumin-to-Creatinine Ratio, bpm: beats per minute. Laboratory units: Hemoglobin (g/dL); Hematocrit (%); Platelet count (×10^3^/μL); White blood cell count (×10^3^/μL); Red blood cell count (×10^6^/μL); Aspartate aminotransferase (AST, U/L); Alanine aminotransferase (ALT, U/L); γ-Glutamyl transpeptidase (U/L); Alkaline phosphatase (U/L); Lactate dehydrogenase (U/L); Total bilirubin (mg/dL); Albumin (g/dL); Creatinine (mg/dL); Uric acid (mg/dL); Fasting blood glucose (mg/dL); HbA1c (%); Total cholesterol (mg/dL); HDL cholesterol (mg/dL); LDL cholesterol (mg/dL); Triglycerides (mg/dL); Sodium (mEq/L); Potassium (mEq/L); Chloride (mEq/L), UACR (mg/gCr).

**Table 2:** Clarke error grid summary for held-out test predictions by model and outcome.

Model	Peak N	Zone A	Zone B	A + B (%)	Nadir N	Zone A	Zone B	A + B (%)
Full-Nutrition	1805	825	612	**79.6**	1805	651	1079	**95.8**
Carbohydrate	1849	877	636	**81.9**	1849	701	1059	**95.2**
Available-Carbohydrate	1849	881	641	**82.3**	1849	715	1044	**95.1**

N denotes the number of held-out test predictions per outcome (per plot: 1805 for Full-Nutrition; 1849 for both Carbohydrate and Available-Carbohydrate). “Zone A” and “Zone B” counts reflect the Clarke error grid clinical accuracy (A = clinically accurate; B = benign error). “A + B (%)” is calculated as 100 × (Zone A + Zone B)/N and reported to one decimal place. Note: Detailed zone counts (C/D/E) are visualized in Figure 2; Table 2 focuses on A/B, which are most relevant for clinical acceptability.

## Data Availability

The de-identified minimal dataset underlying the main findings is available from the corresponding author upon reasonable request, subject to a data use agreement and, where required, institutional approvals (Approval No. RBMR-E-466). The data are not publicly available due to privacy and ethical restrictions associated with time-stamped CGM traces, insulin dosing logs, and meal diaries. The full analysis code (PyTorch preprocessing/training/evaluation scripts) and trained model weights will be made openly available upon acceptance.

## Appendix A

The *y*-axis denotes loss (mean squared error) and the *x*-axis denotes epochs. For the Carbohydrate and Available Carbohydrate models, curves terminate after approximately epoch 115 due to early stopping triggered by the validation criterion. Across models, training loss decreased monotonically, while validation loss initially decreased and then plateaued/slightly increased, indicating the onset of overfitting that was curtailed by early stopping. The loss trajectories for the Carbohydrate and Available Carbohydrate models were nearly superimposed, consistent with the substantial overlap between total and available carbohydrate features in this dataset.

## References

[1] American Diabetes Association (2022). Standards of Medical Care in Diabetes–2022. Diabetes Care.

[2] Davies M.J., D’Alessio D.A., Fradkin J., Kernan W.N., Mathieu C., Mingrone G., Rossing P., Tsapas A., Wexler D.J., Buse J.B. (2018). Management of Hyperglycemia in Type 2 Diabetes, 2018. A Consensus Report by the American Diabetes Association (ADA) and the European Association for the Study of Diabetes (EASD). Diabetes Care.

[3] The Japan Diabetes Society (2024). Diabetes Treatment Guideline 2024.

[4] Inzucchi S.E., Bergenstal R.M., Buse J.B., Diamant M., Ferrannini E., Nauck M., Peters A.L., Tsapas A., Wender R., Matthews D.R. (2015). Management of Hyperglycemia in Type 2 Diabetes, 2015: A Patient-Centered Approach. Update to a Position Statement of the American Diabetes Association and the European Association for the Study of Diabetes. Diabetes Care.

[5] Hirsch I.B., Brownlee M. (2005). Should minimal blood glucose variability become the gold standard of glycemic control?. J. Diabetes Complicat..

[6] Battelino T., Danne T., Bergenstal R.M., Amiel S.A., Beck R., Biester T., Bosi E., Buckingham B.A., Cefalu W.T., Close K.L. (2019). Clinical targets for continuous glucose monitoring data interpretation: Recommendations from the International Consensus on Time in Range. Diabetes Care.

[7] Zhou Z., Sun B., Huang S., Zhu C., Bian M. (2020). Glycemic variability: Adverse clinical outcomes and how to improve it?. Cardiovasc. Diabetol..

[8] Chen J., Yi Q., Wang Y., Wang J., Yu H., Zhang J., Hu M., Xu J., Wu Z., Hou L. (2022). Long-term glycemic variability and risk of adverse health outcomes in patients with diabetes: A systematic review and meta-analysis of cohort studies. Diabetes Res. Clin. Pract..

[9] Heinemann L., Freckmann G., Ehrmann D., Faber-Heinemann G., Guerra S., Waldenmaier D., Hermanns N. (2018). Real-time continuous glucose monitoring in adults with type 1 diabetes and impaired hypoglycaemia awareness (HypoDE): A multicentre, randomised controlled trial. Lancet.

[10] Mougiakakou S.G., Nikita K.S. (2000). A neural network approach to insulin dosing advised by carbohydrate estimation. Diabetes Technol. Ther..

[11] Nasser A.R., Hasan A.M., Humaidi A.J., Alkhayyat A., Alzubaidi L., Fadhel M.A., Santamaría J., Duan Y. (2021). IoT and cloud computing in health-care: A new wearable device and cloud-based deep learning algorithm for monitoring of diabetes. Electronics.

[12] Bao J., Gilbertson H.R., Gray R., Munns D., Howard G., Petocz P., Colagiuri S., Brand-Miller J.C. (2011). Improving the estimation of mealtime insulin dose in adults with type 1 diabetes: The normal insulin demand for dose adjustment (NIDDA) study. Diabetes Care.

[13] Jafar A., Kobayati A., Tsoukas M.A., Haidar A. (2024). Personalized insulin dosing using reinforcement learning for high-fat meals and aerobic exercises in type 1 diabetes: A proof-of-concept trial. Nat. Commun..

[14] Huo L., Deng W., Lan L., Li W., Shaw J.E., Magliano D.J., Ji L. (2022). Real world application of insulin pump therapy among patients with type 1 diabetes in China: A cross sectional study. Front. Endocrinol..

[15] Wang G., Liu X., Ying Z., Yang G., Chen Z., Liu Z., Zhang M., Yan H., Lu Y., Gao Y. (2023). Optimized glycemic control of type 2 diabetes with reinforcement learning: A proof-of-concept trial. Nat. Med..

[16] Daly A.B., Boughton C.K., Nwokolo M., Hartnell S., Wilinska M.E., Cezar A., Evans M.L., Hovorka R. (2023). Fully automated closed-loop insulin delivery in adults with type 2 diabetes: An open-label, single-center, randomized crossover trial. Nat. Med..

[17] Rabby M.F., Tu Y., Hossen M.I., Maida A.S., Hei X. (2021). Stacked LSTM-based deep recurrent neural network with Kalman smoothing for blood glucose prediction. BMC Med. Inform. Decis. Mak..

[18] Shen Y., Kleinberg S. (2025). Personalized blood glucose forecasting from limited CGM data using incrementally retrained LSTM. IEEE Trans. Biomed. Eng..

[19] Li K., Daniels J., Liu C., Herrero P., Georgiou P. (2020). Convolutional recurrent neural networks for glucose prediction. IEEE J. Biomed. Health Inform..

[20] Ahmed B.M., Ali M.E., Masud M.M., Azad M.R., Naznin M. (2024). After-meal blood glucose level prediction for type-2 diabetic patients. Heliyon.

[21] Alkanhel R.I., Saleh H., Elaraby A., Alharbi S., Elmannai H., Alsamhi S.H., Mostafa S. (2024). Hybrid CNN-GRU model for real-time blood glucose forecasting: Enhancing IoT-based diabetes management with AI. Sensors.

[22] Bian Q., As’arry A., Cong X., Rezali K.A., Ahmad R.M.R. (2024). A hybrid Transformer-LSTM model apply to glucose prediction. PLoS ONE.

[23] Moon J., Park S., Chung J.H., Park T., Choi M., Lee S. (2025). Personalized blood glucose prediction in type 1 diabetes using meta-learning with bidirectional LSTM-transformer hybrid model. Sci. Rep..

[24] Karagoz M.A., Breton M.D., El Fathi A. (2025). A comparative study of transformer-based models for multi-horizon blood glucose prediction. arXiv.

[25] Deng Y., Lu L., Aponte L., Angelidi A.M., Novak V., Karniadakis G.E., Mantzoros C.S. (2021). Deep transfer learning and data augmentation improve glucose levels prediction in type 2 diabetes patients. npj Digit. Med..

[26] Veluvali A., Zahedani A.D., Hosseinian A., Aghaeepour N., McLaughlin T., Woodward M., DiTullio A., Hashemi N., Snyder M.P. (2025). Real-world impact of an AI-enhanced continuous glucose monitoring mobile application on glycemic control and weight management. npj Digit. Med..

[27] Brügger L., Kowatsch T., Jovanova M. (2025). Personalized machine learning models for predicting postprandial glucose excursions in Chinese adults with type 2 diabetes. Sci. Rep..

[28] Sheng B., Pushpanathan K., Guan Z., Lim Q.H., Lim Z.W., Yew S.M.E., Goh J.H.L., Bee Y.M., Sabanayagam C., Sevdalis N. (2024). Artificial intelligence in diabetes care: Current applications and future directions. Lancet Diabetes Endocrinol..

[29] Acuna E., Aparicio R., Palomino V. (2023). Analyzing the performance of transformers for the prediction of the blood glucose level considering imputation and smoothing. Big Data Cogn. Comput..

[30] Carvalho C.F., Liang Z. (2024). Glucose prediction with long short term memory (LSTM) models in three distinct populations. Eng. Proc..

[31] Rodríguez Rodríguez I., Chatzigiannakis I., Rodríguez J.V., Maranghi M., Gentili M., Zamora Izquierdo M.Á. (2019). Utility of big data in predicting short term blood glucose levels in type 1 diabetes mellitus through machine learning techniques. Sensors.

[32] Kumnungkit K., Likasiri C., Pongvuthithum R., Tantakitti F. (2022). Universal minimal model for glucose–insulin relationship with the influence of food dynamic. Comput. Math. Methods Med..

[33] Tyler N.S., Mosquera-Lopez C.M., Wilson L.M., Dodier R.H., Branigan D.L., Gabo V.B., Guillot F.H., Hilts W.W., El Youssef J., Castle J.R. (2020). An artificial intelligence decision support system for the management of type 1 diabetes. Nat. Metab..

[34] Jödicke D., Parra D., Kronberger G., Winkler S. (2022). Sparse identification of nonlinear systems for blood glucose prediction using differential equations. arXiv.

[35] Ng E., Kaufman J.M., van Veen L., Fossat Y. (2021). A minimal model of blood glucose homeostasis. arXiv.

[36] Fu S., Li L., Deng S., Zan L., Liu Z. (2016). Effectiveness of advanced carbohydrate counting in type 1 diabetes mellitus: A systematic review and meta-analysis. Sci. Rep..

[37] Vaz E.C., Porfírio G.J.M., Nunes H.R.D.C., Nunes-Nogueira V.D.S. (2018). Effectiveness and safety of carbohydrate counting in the management of adult patients with type 1 diabetes mellitus: A systematic review and meta-analysis. Arch. Endocrinol. Metab..

[38] Bell K.J., Barclay A.W., Petocz P., Colagiuri S., Brand-Miller J.C. (2014). Efficacy of carbohydrate counting in type 1 diabetes: A systematic review and meta-analysis. Lancet Diabetes Endocrinol..

[39] Schmidt S., Schelde B., Nørgaard K. (2014). Effects of advanced carbohydrate counting in patients with type 1 diabetes: A systematic review. Diabet. Med..

[40] Franz M.J. (2004). Carbohydrate counting: A review of its relevance in diabetes management. Diabetes Educ..

[41] Smart C.E., King B.R., McElduff P., Collins C.E. (2012). In children using intensive insulin therapy, a 20-g variation in carbohydrate amount significantly impacts on postprandial glycaemia. Diabet. Med..

[42] Smart C.E., Evans M., O’Connell S.M., McElduff P., Lopez P.E., Jones T.W., Davis E.A., King B.R. (2013). Both dietary protein and fat increase postprandial glucose excursions in children with type 1 diabetes, and the effect is additive. Diabetes Care.

[43] Bell K.J., Smart C.E., Steil G.M., Brand-Miller J.C., King B.R., Wolpert H.A. (2015). Impact of fat, protein, and glycemic index on postprandial glucose control in type 1 diabetes: Implications for intensive diabetes management in the continuous glucose monitoring era. Diabetes Care.

[44] Neu A., Behret F., Braun R., Herrlich S., Liebrich F., Loesch-Binder M., Schumacher U., Schneider A., Schweizer R., Uhlemann M. (2015). Carbohydrate estimation supported by the Diabetes Interactive Diary: Accuracy and usability of bolus calculation. Diabetes Technol. Ther..

[45] Wolever T.M.S., Jenkins D.J.A., Jenkins A.L., Josse R.G. (1991). The glycemic index: Methodology and clinical implications. Am. J. Clin. Nutr..

[46] Nimri R., Dassau E., Segall T., Muller I., Bratina N., Renard E., de Beaufort C., Diem P., Zangen D., Phillip M. (2013). Adjusting insulin doses for fat and protein in children with type 1 diabetes: The Pankowska equation versus food insulin index. J. Diabetes Sci. Technol..

[47] Scidà G., Corrado A., Abuqwider J., Lupoli R., Rainone C., Della Pepa G., Masulli M., Annuzzi G., Bozzetto L. (2024). Postprandial glucose control with different hybrid closed-loop systems according to type of meal in adults with type 1 diabetes. J. Diabetes Sci. Technol..

[48] Bergenstal R.M., Garg S., Weinzimer S.A., Buckingham B.A., Bode B.W., Tamborlane W.V. (2016). Safety of a hybrid closed-loop insulin delivery system in patients with type 1 diabetes. JAMA.

[49] Liu K., Li L., Ma Y., Jiang J., Liu Z., Ye Z., Liu S., Pu C., Chen C., Wan Y. (2023). Machine learning models for blood glucose level prediction in patients with diabetes mellitus: Systematic review and network meta-analysis. JMIR Med. Inform..

[50] Pinsker J.E., Laguna Sanz A.J., Lee J.B., Church M.M., Andre C., Lindsey L.E., Doyle F.J., Dassau E. (2018). Evaluation of an artificial pancreas with enhanced model predictive control and a glucose prediction trust index with unannounced exercise. Diabetes Technol. Ther..

